# Distribution and dynamic changes of Huanglongbing pathogen in its insect vector *Diaphorina citri*

**DOI:** 10.3389/fcimb.2024.1408362

**Published:** 2024-06-13

**Authors:** Chang-Fei Guo, Wei-Zhen Kong, Marguerite Mukangango, Yu-Wei Hu, Yu-Tao Liu, Wen Sang, Bao-Li Qiu

**Affiliations:** ^1^ Engineering Research Center of Biotechnology for Active Substances, Ministry of Education, Chongqing Normal University, Chongqing, China; ^2^ Guangdong Laboratory for Lingnan Modern Agriculture, Guangzhou, China; ^3^ College of Agriculture, Animal Sciences and Veterinary Medicine, University of Rwanda, Butare, Rwanda; ^4^ Key Laboratory of South China Modern Biological Seed Industry, MARA, National Science & Technology Innovation Center for Modern Agricultural Industry, Guangzhou, China

**Keywords:** Huanglongbing, CLAS, *Diaphorina citri*, infection, distribution, dynamic

## Abstract

The Asian citrus psyllid (ACP) *Diaphorina citri* Kuwayama is the leading vector of *Candidatus* Liberibacter asiaticus (*C*Las), the causative agent of citrus Huanglongbing (HLB) disease. The distribution and dynamics of *C*Las within ACP are critical to understanding how the transmission, spread and infection of *C*Las occurs within its host vector in nature. In this study, the distribution and titer changes of *C*Las in various tissues of ACP 5^th^ instar nymphs and adults were examined by *fluorescence in situ hybridization* (FISH) and real-time quantitative PCR (qPCR) techniques. Results demonstrated that 100% of ACP 5^th^ instar nymphs and adults were infected with *C*Las following feeding on infected plants, and that *C*Las had widespread distribution in most of the tissues of ACP. The titers of *C*Las within the midgut, salivary glands and hemolymph tissues were the highest in both 5^th^ instar nymphs and adults. When compared with adults, the titers of *C*Las in these three tissues of 5^th^ instar nymphs were significantly higher, while in the mycetome, ovary and testes they were significantly lower than those of adults. FISH visualization further confirmed these findings. Dynamic analysis of *C*Las demonstrated that it was present across all the developmental ages of ACP adults. There was a discernible upward trend in the presence of *C*Las with advancing age in most tissues of ACP adults, including the midgut, hemolymph, salivary glands, foot, head, cuticula and muscle. Our findings have significant implications for the comprehensive understanding of the transmission, dissemination and infestation of *C*Las, which is of much importance for developing novel strategies to halt the spread of *C*Las, and therefore contribute to the efficient prevention and control of HLB.

## 1 Introduction

The Asian citrus psyllid (ACP) *Diaphorina citri* Kuwayama (Hemiptera: Sternorrhyncha: Psylloidea: Liviidae) is a phloem-feeding insect and major pest of citrus plants (Hall et al., 2013). It is well documented as being the primary insect vector of ‘*Candidatus* Liberibacter asiaticus’ (*C*Las), the bacterial pathogen associated with citrus Huanglongbing (HLB) or citrus greening disease, which is the most serious disease of citrus worldwide (Bové, 2006; Carmo-Sousa et al., 2020). To date, HLB has spread to more than 40 countries in Asia, Oceania, North and South America (Bové, 2006; Croxton and Stansly, 2014; Da Graça et al., 2016). Although research on *C*Las has attracted significant attention, the potential control of *C*Las itself remains largely unknown currently due to the lack of pure cultures of *C*Las, therefore, controlling the insect vector is the most critical step for citrus HLB prevention (Nava et al., 2007; Hall et al., 2013). Given that reducing populations of ACP is critical to HLB management, the biology of the vector and mechanisms of *C*Las acquisition and transmission in ACP hosts must be fully understood.

*C*Las is acquired by ACP nymphs or adults during feeding on the phloem of infected citrus plants and the resulting ingestion of *C*Las into the ACP’s midgut. This is followed by its translocation and probable multiplication into various tissues including the hemolymph and salivary glands, where it is then inoculated into a new plant during feeding (Hall et al., 2013). The ACP nymphs are more efficient at acquiring *C*Las than adults and can transmit HLB efficiently when acquiring *C*Las in the nymphal stage, whereas they are inefficient at transmitting HLB when acquiring *C*Las in the adult stage (Ammar et al., 2011b; Carmo-Sousa et al., 2020). Therefore, it is proposed that *C*Las mainly uses ACP nymphs to acquire pathogens, and ACP adults to inoculate and transmit pathogens (Ammar et al., 2020). When acquired by ACP nymphs, *C*Las is transmitted in a persistent propagative manner with it even accompanying the psyllid throughout its entire life cycle (Inoue et al., 2009; Pelz-Stelinski et al., 2010). During the circulative journey, *C*Las successfully overcomes multiple physiological barriers, including the midgut-infection barrier, dissemination barrier, and salivary-escape barrier. Using quantitative polymerase chain reaction (qPCR), electron microscopy scanning, and *fluorescence in situ hybridization* (FISH), it has been determined that *C*Las is detectable in various ACP tissues including the filter chamber, midgut, hemolymph, salivary glands, ovary, and muscles (Ammar et al., 2011a, b). Hosseinzadeh et al. (2019b) quantified *C*Las titer in multiple tissues of ACP and found that *C*Las were detected in various insect tissues. More recently, several studies have used qPCR to describe *C*Las accumulation in hemolymph and salivary glands, suggesting propagation of *C*Las within insect tissues (Pelz-Stelinski et al., 2010; Ammar et al., 2016; Wu et al., 2018). However, previous studies have primarily focused on the early-stage detection of *C*Las within ACP; limited information is available regarding the distribution and titers of *C*Las among various tissues of an ACP nymph and adult during its development, and indeed the multiplication of *C*Las within various ACP tissues remains poorly understood (Pelz-Stelinski et al., 2010; Ammar et al., 2011a, b, 2016; Hosseinzadeh et al., 2019b; Nian et al., 2023).

Over a prolonged period of co-evolution, an intricate and mutually advantageous symbiotic relationship has evolved among vector insects, pathogenic microorganisms, and receptor plants (Hogenhout et al., 2008); the plant-*C*Las-ACP association system in our current study is also included. Understanding the titers and distribution of pathogens within insects is crucial for elucidating the underlying mechanisms through which pathogenic microorganisms impact the physiological functions of their hosts (Hogenhout et al., 2008; Carmo-Sousa et al., 2020).

It is known that *C*Las multiplies in the host bodies after it acquisition by ACP 5^th^ instar nymph,. however, after the acquisition of *C*Las by ACP adult, the persistence of *C*Las was found to be limited and even eliminated, suggesting that only those ACP adults that were infected *C*Las during their 5^th^ instar nymph stage have high efficiency to transmit *C*Las. The exact mode of infection in different tissues is still unclear to date, to address the knowledge gaps identified above, the presence and distribution of *C*Las in various tissues of an ACP 5^th^ instar nymph and adult were systematically detected through qPCR and FISH. The dynamics of *C*Las in different tissues of an ACP adult was also investigated. The results are expected to aid in further understanding the phenomenon of propagation of *C*Las within the body of an ACP, providing theoretical support for understanding the underlying mechanism behind *C*Las transmission by these insects.

## 2 Materials and methods

### 2.1 Host plants and insects

The colony of ACP was continuously reared at the Guangdong Laboratory for Lingnan Modern Agriculture, Guangzhou, China. The *C*Las-uninfected ACP colony were reared on healthy, *C*Las negative orange jasmine (*Murraya exotica* L.) plants, while the *C*Las positive ACP colony was obtained by inoculating *C*Las-uninfected ACP to a *C*Las-infected tree of *Citrus reticulata* “Shatangju” at Fruit Research Institute of Zhaoqing University (Guangdong, China) for 3 weeks before experiments. Experimental plants were tested periodically as *C*Las-negative or *C*Las-positive by PCR, with nested-PCR also used to ensure the *C*Las infection rate within the ACP as outlined by Guo et al. (2021).

### 2.2 Sample collection and tissue dissection

The 5^th^ instar ACP nymphs were reared on *C*Las-infected Shatangju plants in plant growth chambers (Jiangnan Instrument Co. Ltd., RXZ-380A) at Guangdong Laboratory for Lingnan Modern Agriculture at 26 ± 1°C, RH 80 ± 10% with L:D = 14:10 photoperiods. Following emergence (which was marked as 1 day after eclosion), the *C*Las-infected adults were collected and reared on the *C*Las-uninfected Shatangju plants. Five different developmental stages (1 d, 5 d, 10 d, 15 d and 20 d old) of adults were prepared.

The above collected ACP 5^th^ instar nymphs and adults of different ages were dissected, and various tissues were collected. The hemolymph was collected with a 10 µL pipette tip using the method of Killiny et al (Killiny et al., 2017). For other tissues including midgut, salivary gland, mycetome, ovary, testes, foot, head, cuticula and muscle, dissections were performed in 1×phosphate buffer saline (with 75% RNAfollow All, Yuanye, Shanghai, China, R40036) on ice and collected within a maximum of one hour. After the dissection process, the obtained samples were subsequently divided into two distinct portions to facilitate the conjoint analysis. One portion was utilized for FISH in order to detect the distribution of *C*Las, while the other portion was allocated for qPCR to determine the relative titers of *C*Las. Three biological replications were conducted, wherein each replication consisted of 30–40 individuals for qPCR analysis; 5 tissue samples including midgut, salivary glands, testis, and ovary of ACP 5^th^ instar nymphs and adults were imaged in FISH detection. Five replications for each tissue type were repeated to verify the repeatability.

### 2.3 DNA extraction of ACP

DNA extraction of ACP: Each isolated tissue, including midgut, salivary glands, head, muscle, cuticula, foot, mycetome, ovary and testes, was washed twice in 1×PBS and each time was 5 minutes, afterward, the tissues and collected hemolymph were transferred to 50µL GA buffer lysate using a dissecting needle. The DNA extraction of the different ACP tissues were followed the instructions of the TIANamp Micro DNA Kit (Tiangen, Beijing, China, DP316).

### 2.4 FISH detection of *C*Las

FISH detection of *C*Las: In order to gain further insights into the distribution of *C*Las within the main tissues, FISH determination was used to visualize the presence of *C*Las within the anatomical structures of the midgut, salivary gland, ovary and testis of the ACP 5^th^ instar nymphs and adults following the protocol of Guo et al. (2023). After a series of fixation, washing, decolorization and hybridization, the nuclei of dissected tissues were stained with DAPI (4’, 6-diamidino-2-phenylindole; 0.1 mg/mL; ThermoFisher Scientific, Waltham, MA, USA, 62248) for 10 min and then rinsed with PBST for 10 min. The stained samples were then observed and photographed under a Nikon Eclipse Ti-U inverted microscope (Nikon Instruments Inc., Tokyo, Japan). The *C*Las probe 5’-Cy3-GCCTCGCGACTTCGCAACCCAT-3’ (targeting the 16S rDNA gene of *C*Las) was labeled at the 5’-terminal with fluorescence labeling of Cy3.

### 2.5 Dynamics detection of *C*Las in different ACP tissues

The titer of *C*Las in different tissues of ACP, including midgut, salivary glands, hemolymph, mycetome, ovary, head, foot, cuticula, muscle and fat body, at different developmental times (1d, 5d, 10d, 15d and 20d) were detected using qPCR. The *C*Las primers (targeting *C*Las prophage genes) used for qPCR were F: 5’-GCCGTTTTAACACAAAAGATGAATATC-3’, R: 5’-ATAAATCAATTTGTTCTAGTTTACGAC-3’ (Morgan et al., 2012), and the *β-Actin* gene of ACP was chosen as a reference gene for the purpose of normalizing and quantifying the data: whose primers were F: 5’-CCCTGGACTTTGAACAGGAAA-3’, R: 5’-CTCGTGGATAC CG CAAGATT-3’ (Tiwari et al., 2012). The reaction system of qPCR was as follows: 5 µL SYBR Premix Ex Taq (Takara, Dalian, China, RR820A), 2 µL DNA template, 0.5 µL (10 µmol/L) forward/reverse primer and 2 µL ddH_2_O, at a total reaction volume of 10 µL. The reactions were conducted using a Bio-Rad CFX Co-nnect™ thermocycler (Bio-Rad Laboratories Inc., Hercules, CA, USA) according to the following protocol: initial denaturation at 95°C for 3 min, followed by 40 cycles at 95°C for 10 s, 60°C for 20 s and 72°C for 30 s. The relative titers of *C*Las (relative expression of *C*Las) was calculated using the 2^-ΔΔCt^ method (Livak and Schmittgen, 2001).

### 2.6 Detection of *C*Las in ACP saliva

The *C*Las in the saliva secreted by the different stages (1 d, 5 d, 10 d, 15 d and 20 d) of *C*Las-infected ACP adults was detected by nested PCR. Thirty *C*Las-infected ACP adults were collected and fed with artificial diet (containing 15% sucrose solution, 0.1% green food dyes and 0.4% yellow food dyes) using the Parafilm double-film method (Galdeano et al., 2017). The ACP adults without *C*Las were used as a parallel control. Following 3 days of feeding, the genomic DNA was extracted from the artificial diet using a micro-scale genomic DNA extraction kit (Tiangen, Beijing, China, DP316) for *C*Las detection, following the instructions provided with the kit for “Extraction of Genomic DNA from Mouthwash”. The template DNA was amplified using a rolling circle amplification (RCA) kit (Haigene, Haerbin, China, A3702a) to increase its concentration, and then nested PCR was used to detect *C*Las within the saliva.

The primer sequences for nested PCR (targeting the *β-operon* gene of *C*Las) were P1, P2, P3 and P4 (P1: 5’-TCTGTTTTCTTCGAGGTTGGTGAG-3’, P2: 5’-ACCGCAAGACTCCTTACCAG GAAG-3’, P3: 5’-GCGTTCATGTAGAAGTTGTG-3’, P4: 5’-CTTACAGGTGGCTGACTCAT-3’). The first round of PCR reaction included 12 μL Taq enzyme mix (Mei5bio, Beijing, China, MF002-plus-10), 10 μL ddH_2_O, 1 μL each of P1 and P2 primers and 1 μL DNA template. The PCR protocol consisted of an initial denaturation step at 94°C for 5 minutes, followed by 20 cycles of denaturation at 94°C for 30 seconds, annealing at 50°C for 30 seconds, and extension at 72°C for 1 minute, with a final extension step at 72°C for 5 minutes. The second round of PCR reaction was comprised of 12 μL Taq enzyme mix, 9 μL ddH_2_O, 1 μL each of the P3 and P4 primers (10 μmol/L) and 2 μL of DNA template (i.e., the first-round PCR product). The PCR program included an initial denaturation step at 94°C for 5 min, followed by 35 cycles of denaturation at 94°C for 30s, annealing at 50°C for 30s, and extension at 72°C for 30s. A final extension step at 72°C lasting five minutes concluded the PCR process.

### 2.7 Statistical analysis

Relative titer of *C*Las in different tissues of ACP were calculated using the method of 2^-ΔΔCt^. ACP β-Actin was used as the internal control for normalization. The column and error bars represent the fold change (titer) in Mean ± SE. Different letters indicate significant differences in *C*Las gene expression among different treatments (P <0.05).

In order to comprehensively understand the copy number of *C*Las within an infected ACP, we took into account the scenario where the Ct*_Actin_* value exceeded the Ct*_C_*_Las_ value by employing the formula-POWER (2, (Ct_Actin_-Ct*_C_*_Las_)) to calculate the relative number of *C*Las within individual cells of various tissues, where Ct*_C_*_Las_ is the cycle threshold of the gene of *C*Las and Ct*_Actin_* is the cycle threshold value of the housekeeping gene (*β-actin*). If the *C*Las content within a single cell surpasses the copy number of the reference *Actin* gene, this means the *C*Las content within a single cell > 1.0.

All fluorescent images were prepared for final presentation using Adobe Photoshop CS5. It is imperative to assess the normality and homogeneity of variance for all the data prior to utilization. The data analyses were conducted utilizing SPSS 18.0 software (SPSS Inc., Chicago, IL, USA). Statistical analysis was performed via Student’s t-test or One-Way Analysis of Variance (ANOVA). Graphs were drawn using GraphPad Prism 6.0 and Microsoft Excel software.

## 3 Results

### 3.1 *C*Las infection in ACP 5^th^ instar nymphs and adults

To assess the acquisition rate of *C*Las by ACP, *C*Las-free ACP adults were introduced to oviposit on the *C*Las-infected citrus plants. After the progeny developed through to 5^th^ instar nymphs and teneral adults, the infection rate of *C*Las was detected. Results demonstrated that 100% of 5^th^ instar nymphs and adults were infected with *C*Las when they developed on *C*Las-infected citrus plants (**Table 1**); indicating a high ability of ACP nymphs to acquire *C*Las.

**Table 1:** Infection rates of *C*Las in ACP 5^th^ instar nymphs and teneral adults.

Developmental stage	Number of tests	Number of infections	Infection rate (%)
5^th^ instar nymph	20	20	100%
Teneral adult	20	20	100%

### 3.2 *C*Las titers in different tissues of 5^th^ instar nymphs and teneral adults

**Figure 1:**
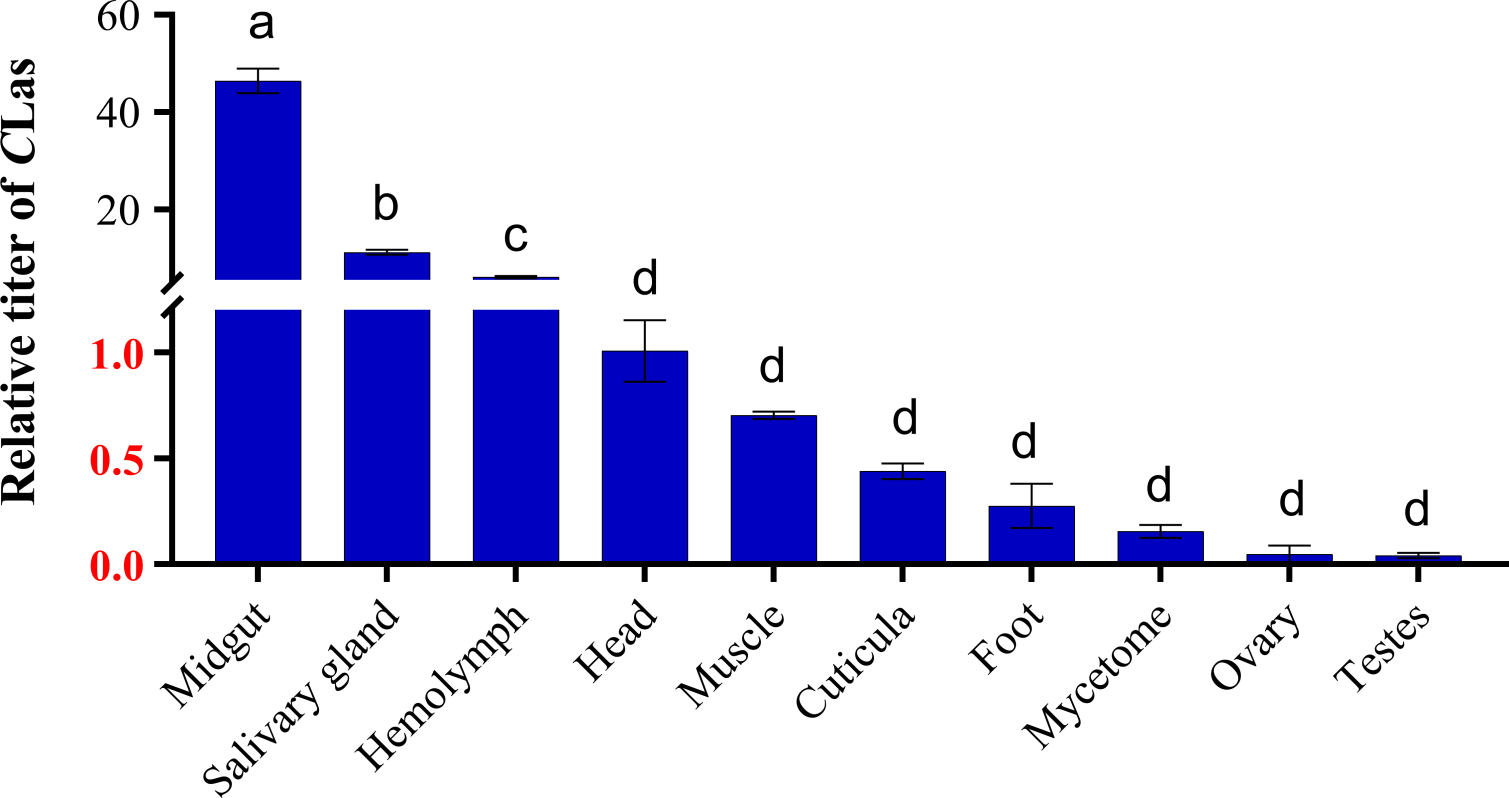
Relative titer of *C*Las in different tissues of ACP 5^th^ instar nymphs. ACP *β-Actin* was used as the internal control for normalization. The relative titers were calculated using the method of 2^-ΔΔCt^. The relative titer of *C*Las in other tissues is relative to the titer in the head. The column and error bars represent the fold change (titer) in Mean ± SE. Different letters indicate significant differences in *C*Las gene expression among different treatments (*P <*0.05).

The relative titers of *C*Las within *C*Las-infected 5^th^ instar nymphs and adults were investigated by qPCR. The results demonstrated that *C*Las infested all the tissues, including midgut, salivary gland, hemolymph, head, muscle, cuticula, foot, mycetome, ovary and testes. In addition, midgut, salivary glands and hemolymph were the tissues with the highest titers of *C*Las; the titer of *C*Las in the midgut was 4.1 times higher than that in the salivary glands, and 7.6 times higher than in the hemolymph (**Figure 1**).

**Figure 2:**
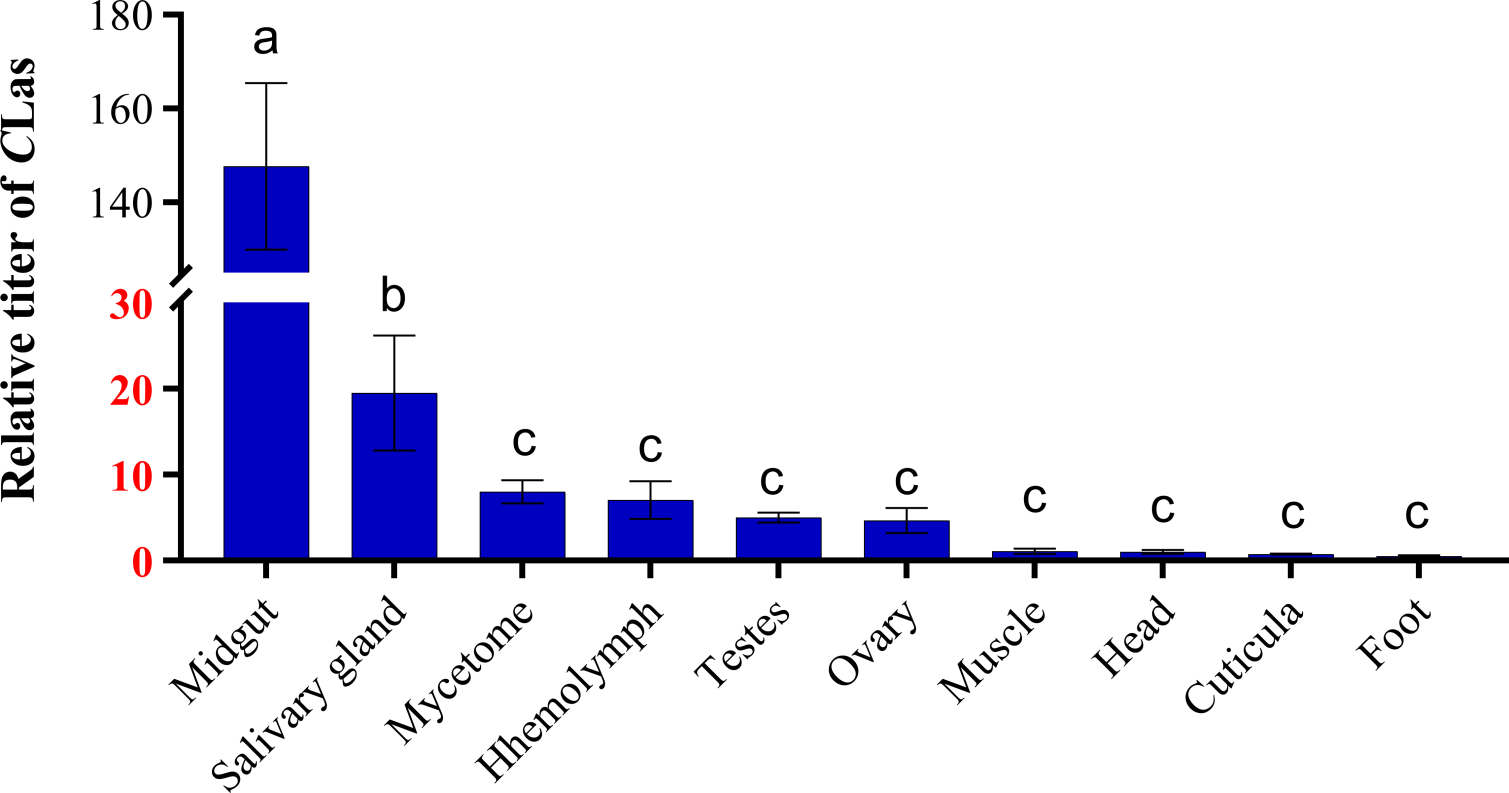
Relative titer of *C*Las in different tissues of ACP teneral adults. ACP *β-Actin* was used as the internal control for normalization. The relative titers were calculated using the method of 2^-ΔΔCt^. The relative titer of *C*Las in other tissues is relative to the titer in the muscle. The column and error bars represent the fold change (titer) in Mean ± SE. Different letters indicate significant differences in *C*Las gene expression among different treatments (*P <*0.05).

Similarly, *C*Las was detected in all tissues of teneral adults. Among these tissues, the midgut and salivary glands exhibited higher levels of *C*Las, with the midgut displaying the highest concentration, surpassing that of the salivary glands by a factor of 7.6. Furthermore, the concentration of *C*Las in the midgut was 18.5 times higher than that in the mycetome, and 21.1 times higher than that in the hemolymph (**Figure 2**). Conversely, lower titers of *C*Las were detected in the head, muscle, cuticula, foot, ovary and testes.

### 3.3 Titer comparison of *C*Las in different tissues of 5^th^ instar nymphs and teneral adults

**Figure 3:**
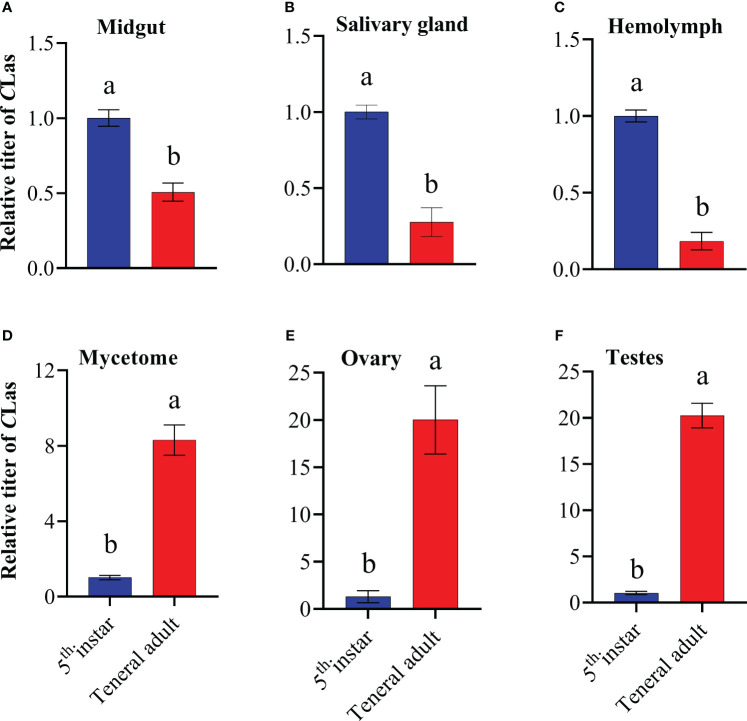
Relative titer of *C*Las in different tissues of ACP 5^th^ instar nymphs and teneral adults. **(A)** midgut; **(B)** hemolymph; **(C)** salivary gland; **(D)** mycetome; **(E)** ovary; **(F)** testes. ACP *β-Actin* was used as the internal control for normalization. The relative titers were calculated using the method of 2^-ΔΔCt^. The relative titer of *C*Las in adults is relative to the titer in nymphs. The column and error bars represent the fold change (titer) in Mean ± SE. Different letters indicate significant differences in *C*Las gene expression among different treatments P <0.05 (independent-sample t-test).

To comprehend the alterations of *C*Las titers in different tissues of 5^th^ instar nymphs and adults, the titers of *C*Las in the midgut, salivary glands, hemolymph, mycetome, ovary and testes were detected using qPCR. Results revealed a significant disparity in *C*Las titers between the 5^th^ instar nymphs and adults, for example, the *C*Las titers in the midgut, salivary glands and hemolymph of 5^th^ instar nymphs were significantly higher than those in adults, while the mycetome, ovary, and testes of 5^th^ instar nymphs exhibited significantly lower *C*Las titers compared to that of teneral adults (**Figure 3**), which suggests that during the development from nymph to adult, the content of *C*Las in different tissues undergoes different trends of change.

### 3.4 *C*Las distribution in the main tissues of 5^th^ instar nymphs and adults

**Figure 4:**
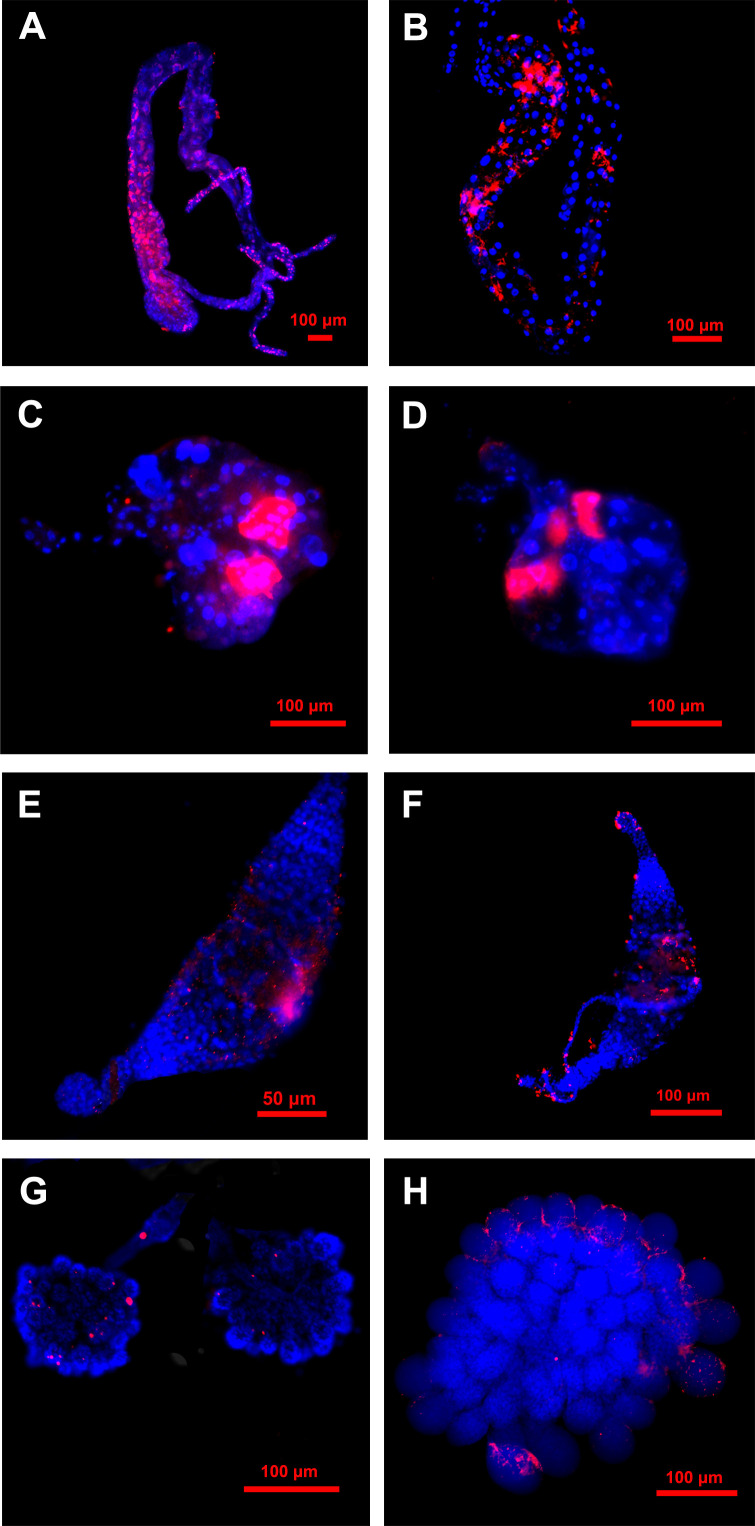
Localization of *C*Las in different tissues of 5^th^ instar nymphs and adults of ACP. **(A)** midgut of 5^th^ instar nymph; **(B)** midgut of adult; **(C)** salivary gland of 5^th^ instar nymph; **(D)** salivary glands of adult; **(E)** testes of 5th instar nymph; **(F)** testes of the adult;**(G)** ovary of 5^th^ instar nymph; **(H)** ovary of adult. Nuclear was stained with DAPI (blue); *C*Las was stained with *C*Las-Cy3 probe (red).

Based on the aforementioned result, it is evident that the midgut and salivary glands of the ACP serve as the primary sites of *C*Las infection, there by acting as significant barriers to the transmission of *C*Las, additionally, the ovaries and testis also were infected with *C*Las in moderate quantities. The FISH assay results demonstrated that the fluorescence signals of *C*Las in the midgut were more pronounced in 5^th^ instar nymphs compared to teneral adults (**Figures 4A, B**), which was consistent with the *C*Las titer results of qPCR analysis (**Figure 3A**), which indicated a higher *C*Las titer in the filter chamber. *C*Las fluorescence signals were both found in the salivary glands of 5^th^ instar nymphs and teneral adults. *C*Las was exclusively localized within particular cells of the salivary glands of nymphs and adults. Additionally, the salivary glands of 5^th^ instar nymphs exhibited stronger *C*Las fluorescence signals compared to those of teneral adults (**Figures 4C, D**).

The fluorescence signals of *C*Las in the testes of 5^th^ instar nymphs and teneral adults exhibited diminished intensity and a dispersed pattern (**Figures 4E, F**). The fluorescence signals of *C*Las in the ovaries of 5^th^ instar nymphs and adults were also relatively weak, with only a few discrete fluorescence signals observed (**Figures 4G, H**). However, in the ovaries of teneral adults, it was evident that *C*Las primarily localized around the oocyte, while only a limited number of *C*Las infiltrated the interior (**Figure 4H**). In contrast, the fluorescent signal of *C*Las was not detected in the *C*Las-negative control group (**Figure S1**).

### 3.5 Dynamics of *C*Las multiplication in the main tissues of adult ACP

**Figure 5:**
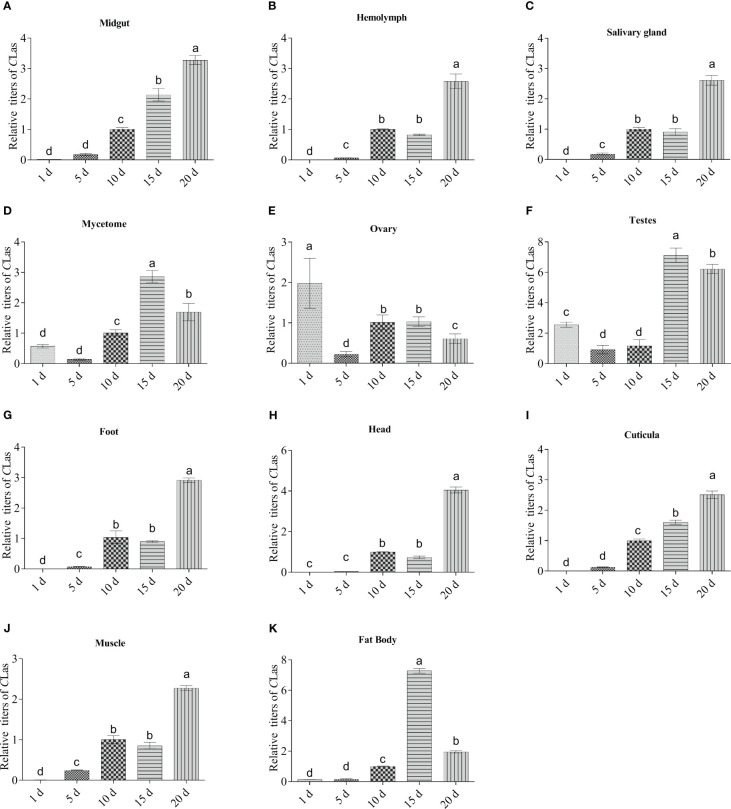
Dynamics of *C*Las multiplication in eleven tissues of adult ACP. **(A)** midgut; **(B)** hemolymph; **(C)** salivary gland; **(D)** mycetome; **(E)** ovary; **(F)** testes; **(G)** foot; **(H)** head; **(I)** cuticula; **(J)** muscle; **(K)** fat body. ACP *β-Actin* was used as the internal control for normalization. The relative titers were calculated using the method of 2^-ΔΔCt^. The column and error bars represent the fold change (titer) in Mean ± SE. Different letters indicate significant differences in *C*Las gene expression among different treatments (*P <*0.05).

The relative titers of *C*Las in various tissues of infected ACP spanning a period of 1 to 20 days from emergence is shown in **Figure 5**. Results demonstrated that *C*Las was presented across all the developmental ages of ACP adults tested, and that its relative titers varied among different tissues. In general, there was a discernible upward trend in the presence of *C*Las with advancing age in most tissues of ACP adults, including midgut, hemolymph, salivary glands, foot, head, cuticula and muscle, which suggests that *C*Las has the ability to propagate and reproduce within these specific anatomical structures (**Figures 5A-C, G-J**). In mycetome, ovary and testes, the *C*Las titers showed a trend of decreasing-increasing-f-decreasing (**Figures 5D–F**), but the *C*Las titers in mycetome and testes on days 15 and 20 were significantly higher than those of days (1, 5 and 10) (**Figures 5D, F**), while the *C*Las titers in an adult ovary on day (5, 10, 15 and 20) were significantly lower than that of day 1 (**Figure 5E**).

### 3.6 Detection of *C*Las in the saliva of ACP adults at different development stages

**Figure 6:**
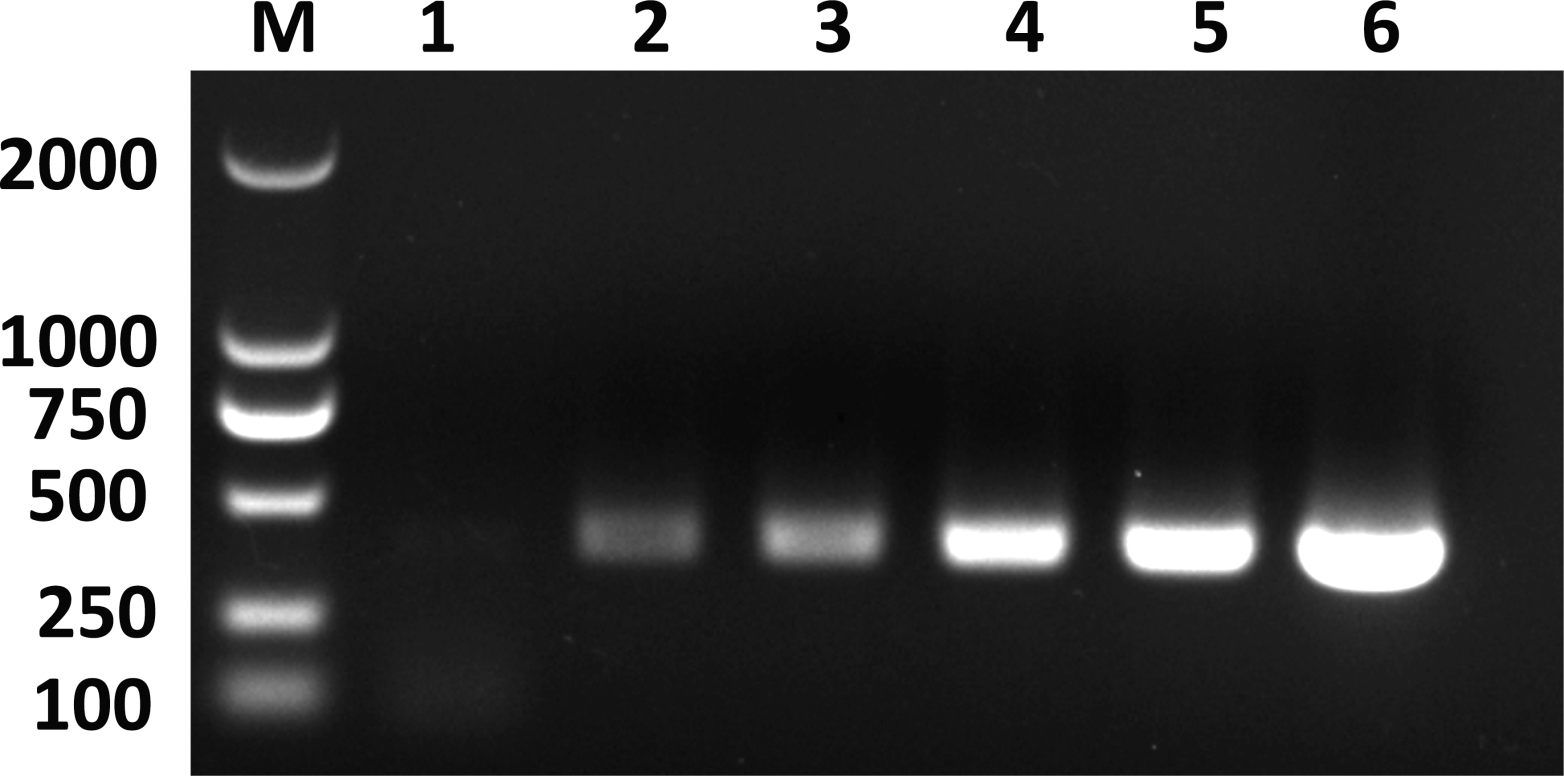
*C*Las detection in the saliva of citrus psyllid. *C*Las in ACP saliva was detected using nested PCR. M: DNA marker; Lane 1: saliva of *C*Las-free ACP, Lanes 2–6: saliva of ACP adults on day 1, 5, 10, 15, 20.

The gel results demonstrated that *C*Las was detected in the saliva of *C*Las- infected ACP at various developmental stages (**Figure 6**). In addition, the PCR bands exhibited a progressive enhancement as the age of the ACP increased, thereby suggesting a corresponding increase in the transmission capacity of *C*Las by the ACP with advancing age.

### 3.7 Relative copy of *C*Las in cells of each tissue compared to *Actin*

**Figure 7:**
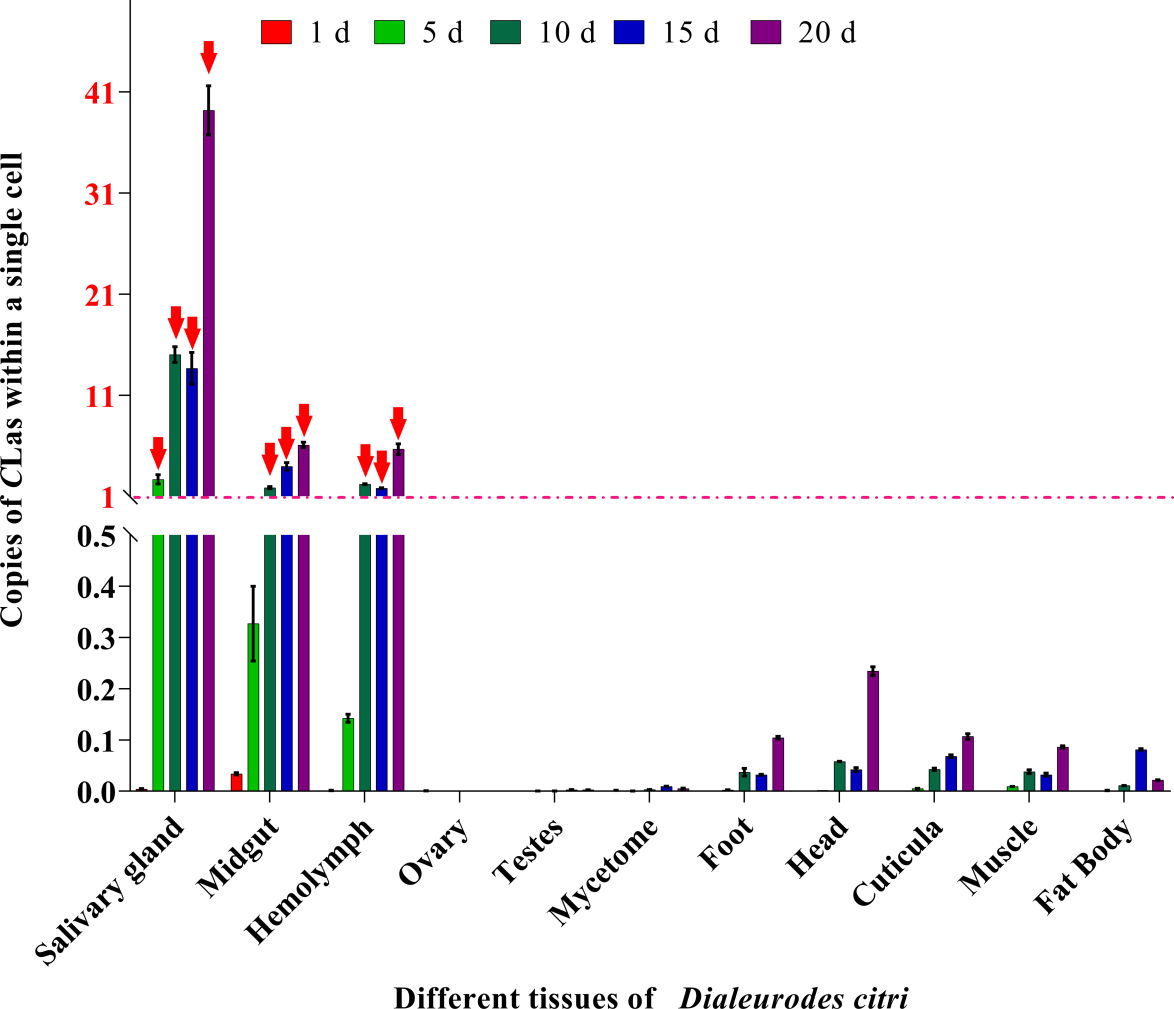
Relative copies of *C*Las in cells of each tissue compared to ACP *β-Actin* gene. The formula POWER (2, (Ct*_Actin_*- Ct*_C_*_Las_)) was used to calculate the relative number of *C*Las in a single cell of each tissue. When the value is greater than 1, it indicates that the tissue has more than 1 unit *C*Las within a single cell. Red arrowheads: The value of relative copies of CLas is greater than 1.

The relative copy of *C*Las and *Actin* genes in the cells of different tissues of ACP are compared in **Figure 7**. Results showed that the values of *C*Las in the salivary glands (days 5–20), midgut (days 10–20) and hemolymph (days 10–20) were all > 1.0, which indicated relative higher amounts of *C*Las in the individual cells of these three tissues. Conversely, the remaining tissues (ovary, testes, mycetome, foot, head, cuticula, muscle and fat body) displayed a relatively lower amount of *C*Las in single cells, consistent with our other results. These observations further support the notion that the salivary glands, midgut and hemolymph serve as the primary sites for *C*Las proliferation and replication.

## 4 Discussion

Citrus Huanglongbing (HLB) is a serious disease of citrus and has caused destructive loss to the citrus industry worldwide for many decades (Bové, 2006; Carmo-Sousa et al., 2020). As the pathogen of HLB, *C*Las is transmitted by ACP in a persistent and propagative manner, completing its circulate transmission from *C*Las-positive plants to healthy plants (Ammar et al., 2016). The transmission mechanism consists of multiple phases, which encompass: (a) the ingestion of the pathogen and its migration into the midgut of ACP while feeding on an *C*Las-infected citrus plant, (b) the transfer of *C*Las from the midgut into the hemolymph and different tissues, including the salivary glands, with potential pathogen replication in these tissues, and (c) the introduction of the *C*Las through injection within salivary secretions during subsequent feeding on a healthy plant that is susceptible to infection (Hogenhout et al., 2008; Mercer et al., 2010; Galdeano et al., 2020). Over the past decade, the acquisition of *C*Las by ACP has been found to be a significant factor in pathogen transmission, and it was influenced by various factors such as temperature, citrus susceptibility, life stage, the sex of the psyllid, season, sampling method, time of acquisition and plant species, resulting in infection rates ranging from 1 to 90% (Inoue et al., 2009; Pelz-Stelinski et al., 2010; Perilla-Henao and Casteel, 2016; Wu et al., 2018). However, our findings revealed a 100% infection rate among ACP. The observed high infection rate could potentially be attributed to the continuous consumption of *C*Las-infected citrus plants by the ACP population under a laboratory experimental design. This prolonged feeding behavior provided an advantageous circumstance for *C*Las to effectively infiltrate all tissues, thereby reinforcing the notion that the nymph stage of ACP exhibits a notable proficiency in acquiring *C*Las (Pelz-Stelinski et al., 2010; Ammar et al., 2016).

Understanding the impact of infection titer and distribution of *C*Las in ACP is of utmost significance. Previously, *C*Las has been identified in the salivary glands, alimentary canal, and overall body of ACP adults through the utilization of qPCR (Ammar et al., 2011b). Additional investigations employing FISH have demonstrated the presence of *C*Las in most tissues of ACP, in addition to those previously mentioned (Ammar et al., 2011a; Coletta et al., 2014; Ammar et al., 2019; Nian et al., 2023). Most of the prior research primarily focused on the detection *C*Las infection in limited number of tissues of ACP adults, neglecting *C*Las infection in different tissues of ACP 5^th^ instar nymphs and lacking a thorough and systematic examination of the infection pattern of *C*Las in various tissues of ACP adults at different instars. In the present study, we conducted a thorough examination of various tissues of ACP 5^th^ instar nymphs and adults to detect *C*Las for the first time, and it was observed that *C*Las also exhibited a systemic invasion in all dissected tissues of an ACP 5^th^ instar nymphs and adults, including the midgut, salivary gland, hemolymph, head, muscle, cuticula, foot, mycetome, ovary and testes. Our findings indicated that *C*Las has the ability to infest various tissues of the ACP in a systematic manner when acquired during the ACP 5^th^ instar nymphs, thus supporting the hypothesis that the ACP 5^th^ instar nymph is proficient in disseminating and acquiring *C*las than adult (Ammar et al., 2011b, 2020; Carmo-Sousa et al., 2020). Our study also identified notable variations in the *C*Las titers attributed to the life stage of ACP, wherein the titer of *C*Las in the midgut, salivary glands, and hemolymph of ACP 5^th^ instar nymphs was significantly higher compared to that of adults. The significantly higher efficiency of *C*Las acquisition by ACP nymphs compared to adults, is possibly due to their higher frequency and longer duration of phloem feeding (Inoue et al., 2009; Pelz-Stelinski et al., 2010; Ammar et al., 2016; Mann et al., 2018). However, it is also important to consider that successful acquisition may be influenced by other factors such as disparities in innate immunity between nymphs and adults (Mann et al., 2011; Ghanim et al., 2017; George et al., 2018).

Two tissues, midgut and salivary glands, are recognized as the most important transmission barriers of persistently and propagatively transmitted plant pathogens (Ramsey et al., 2017; Mishra and Ghanim, 2022). During *C*Las infection, the pathogen must traverse the intestinal barrier or evade immune responses in order to reach the salivary glands for efficient transmission. Ammar et al. observed that the levels of *C*Las in the midgut and salivary glands of ACP adults were considerably greater compared to those in other tissues (Ammar et al., 2011a, b). In the current study, similar findings were observed in 5^th^ instar nymphs and teneral adults; i.e. significantly greater titers of *C*Las in the midgut and salivary glands compared to other bodily tissues. Additionally, our study also demonstrated that the salivary glands (days 5–20), midgut (days 10–20) and hemolymph (days 10–20) exhibited a significantly elevated copy number of *C*Las in individual cells (**Figure 7**), consistent with the results that the relative copy number of *C*Las genomes was significantly higher in both midgut and salivary glands compared to the reference genes of ACP genomes in each sample (Ammar et al., 2011b). The elevated concentration of *C*Las in the hemolymph may increase the likelihood of its infection in the salivary glands.

From another perspective, our results align with previous studies on vector insects being responsible for transmitting plant pathogens and confirmed that the midgut and salivary glands of insects serve as a significant impediment for pathogen transmission (Hogenhout et al., 2008; Wang et al., 2023). A significant indication of the transmission capability of an insect vector related to plant pathogens, is the ability of the pathogen to infiltrate particular cells within the salivary glands of vector insects, and amass in substantial quantities, thereby attaining a sufficient concentration to facilitate transmission of the plant pathogen (Weintraub and Beanland, 2005; Galdeano et al., 2020; Wang et al., 2023).

In this study, the FISH technique was employed to ascertain that *C*Las was exclusively localized within particular cells of the salivary glands of 5^th^ instar nymphs and adults of ACP. The results, combined with qPCR data, indicate that the salivary glands exhibited a higher accumulation of *C*Las compared to other tissues (excluding the midgut). By conducting further testing, it was discovered that the titer of *C*Las within the saliva of the ACP adults gradually increased with age. Consequently, it can be inferred that ACP adults possess the ability to transmit *C*Las during various developmental stages.

Previous studies have revealed the infrequent infection of *C*Las in the ovaries of ACP through qPCR (Weintraub and Beanland, 2005; Ammar et al., 2011a, b). Hosseinzadeh et al. further observed occasional existence of *C*Las in the follicle cells of females (Hosseinzadeh et al., 2019b). In the current study, the presence of *C*Las was also identified in ovary tissues through the utilization of qPCR and FISH, albeit at a low level. It is worth noting that *C*Las exhibited predominant localization around the oocyte in the ovaries of ACP adults, with only a minimal infiltration into the oocyte, which may provide a possible explanation that usually extremely low *C*Las can be maternally transmitted to the next generation (Ghanim et al., 2017; Carmo-Sousa et al., 2020).

The transmission dynamics of *C*Las in ACP suggest a durable association between the pathogen and vector, suggesting that the bacterium responsible for HLB circulates, infects, and potentially replicates within multiple tissues of the vector (Pelz-Stelinski et al., 2010; Wu et al., 2018; Hosseinzadeh et al., 2019b; Nian et al., 2023), which has received constant attention. For instance, Wu et al. reported that *C*Las multiplication was detected in the hemolymph and salivary glands of ACP adults after the bacterium was acquired by nymphs (Wu et al., 2018). Nian et al. reported that the relative *C*Las titer in the midgut and ovaries obviously increased with development (Nian et al., 2023). Based on this premise, we conducted a more detailed detection of the *C*Las infection pattern in various tissues of ACP at different developmental stages. In the present study, it was found that the relative titer in midgut, hemolymph, salivary glands, foot, head, cuticula, and muscle were of a discernible upward trend with advancing age. This observation implies that *C*Las possesses the capacity to propagate and reproduce within these particular anatomical structures. Alternatively, the fluctuation in *C*Las titer observed in the mycetome cells and fat body of ACP may potentially be associated with endosymbiosis and immune responses within the insect (Hosseinzadeh et al., 2019b).

Previous work has found that vitellogenin-like protein (Vg-VWD) in ACP interacts with a *C*Las flagellum (flaA) protein, which is upregulated in *C*Las-infected ACP (Hosseinzadeh et al., 2019a). This implies that the presence of an adequate quantity of Vg within the ACP facilitates the binding of *C*Las. As widely acknowledged, vitellogenin (*Vg*) is expressed in the fat body in an insect, secreted into the hemolymph, and then taken up by developing oocytes (Hosseinzadeh et al., 2019a). According to our previous study (Tufail et al., 2014), the fecundity peak appeared on approximately day 15. Consequently, it is necessary for the fat body to increase its production of Vg during this specific period. However, the titer of *C*Las in the ovary exhibited a gradual decline, whereas the *C*Las titer in the testes initially decreased and subsequently increased after day 15. Whatever the case may be, it is noteworthy that the *C*Las titer in the ovary and testes were comparatively lower when compared to other tissues (**Figure 2**), which also provides a possible explanation that extremely low levels of *C*Las can be maternally transmitted to progeny (Ren et al., 2016; Ghanim et al., 2017). Therefore, regulation of *Vg*-related immune pathways may serve as a mechanism to modulate the vector competence of ACP for *C*Las, suggesting a potential role for elevated Vg expression in females as a defense against ovary infection by *C*Las. Nevertheless, these hypotheses necessitate additional empirical verification.

In conclusion, this study reveals that during the nymph stage, ACP can acquire *C*Las by feeding on a *C*Las-infected citrus plant with high efficiency, and then *C*Las consequently reaches various tissues of ACP in a systematic and comprehensive way, so revealing its ability to replicate or accumulate in the diverse tissues of ACP hosts including midgut, hemolymph, salivary glands, foot, head, cuticula, and muscle. These results aid in comprehensively understanding the different dynamic changes of titers in different tissues of ACP, which is important for developing in-depth research and innovation towards better “*C*Las transmission blocking” strategies for HLB management.

## Data available statement

The original contributions presented in the study are included in the article/**Supplementary Material**. Further inquiries can be directed to the corresponding author.

## Ethics statement

The manuscript presents research on animals that do not require ethical approval for their study.

## Author contributions

CG: Conceptualization, Formal analysis, Investigation, Methodology, Software, Writing – original draft, Writing – review & editing. WK: Investigation, Methodology, Software, Writing – original draft, Writing – review & editing. MM: Conceptualization, Methodology, Writing – review & editing. YH: Conceptualization, Investigation, Resources, Writing – review & editing. YL: Conceptualization, Resources, Supervision, Writing – review & editing. WS: Conceptualization, Methodology, Supervision, Writing – review & editing. BQ: Funding acquisition, Resources, Supervision, Writing – original draft, Writing – review & editing.

## Supplementary material

Supplementary Figure 1 Negative control for the Localization of *C*Las in different tissues of 5^th^ instar nymphs and adults of ACP. **(A)** midgut of 5^th^ instar nymph; **(B)** midgut of adult; **(C)** salivary gland of 5^th^ instar nymph; **(D)** salivary glands of adult; **(E)** testes of 5th instar nymph; **(F)** testes of the adult;**(G)** ovary of 5^th^ instar nymph; **(H)** ovary of adult. Nuclear was stained with DAPI (blue); *C*Las was stained with *C*Las-Cy3 probe (red).
