# Supplementary figures and images for: Distribution and dynamic changes of Huanglongbing pathogen in its insect vector Diaphorina citri
Source: Front Cell Infect Microbiol. 2024 Jun 13;14:1408362. doi: 10.3389/fcimb.2024.1408362 (PMC11208332; doi:10.3389/fcimb.2024.1408362)

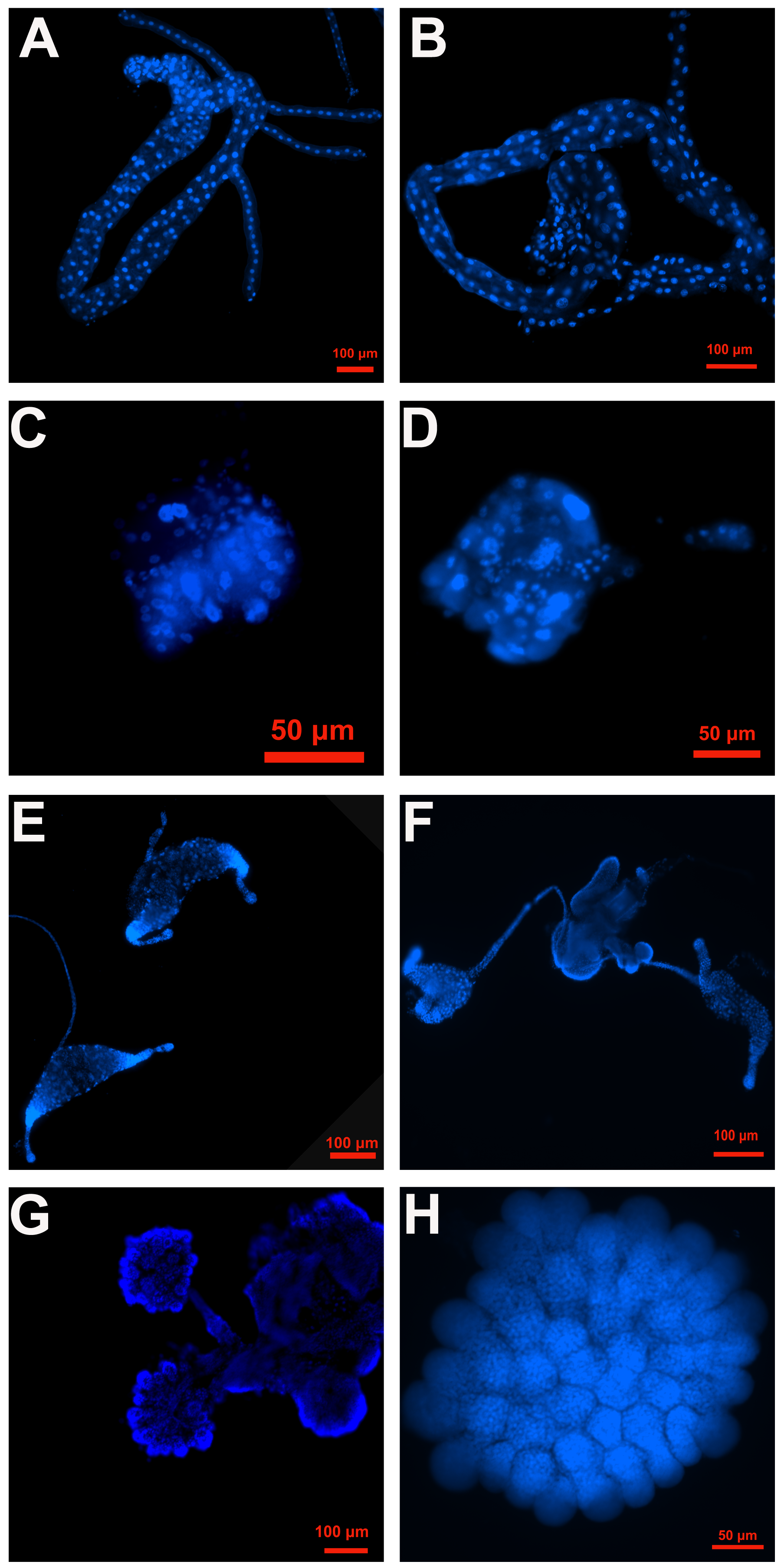

Supplement: Supplementary Figure 1 — Negative control for the Localization of CLas in different tissues of 5th instar nymphs and adults of ACP. (A) midgut of 5th instar nymph; (B) midgut of adult; (C) salivary gland of 5th instar nymph; (D) salivary glands of adult; (E) testes of 5th instar nymph; (F) testes of the adult;(G) ovary of 5th instar nymph; (H) ovary of adult. Nuclear was stained with DAPI (blue); CLas was stained with CLas-Cy3 probe (red). [file Image_1.tif]
